# Biography Contemporary Physicians of Texas

**Published:** 1889-12

**Authors:** 


THE BIOGRAPHY OF CONTEMPORARY PHYSICIANS OF TEXAS is shortly to be published in this city by the Texas Biographical Publishing Company, in connection with "Types of Successful Men of Texas" under the same cover, consent to the said consolidation having been obtained of the majority of the subscribers to the former. The work is now in press and will be issued without delay, as soon as it can be printed and bound. It will be a grand work, of perhaps 800 pages, on heavy paper, and in Morocco and gilt. It will contain much of private and public history never before published, illustrated with seventy-five fine portraits, embracing representatives of every class, statesmen, physicians, jurists, divines, farmers, bankers, merchants, ranchmen, etc. Not the least interesting feature will be a history of the Texas State Medical Association, from its organization to date, from the chaste and eloquent pen of Dr. R. H. L. Bibb, of Saltillo, Mexico, late Secretary of said Association. The subscription list is closed as to the insertion of further sketches, but subscriptions for the book, to a very limited number, will be received; price, ten dollars each. The sketches will all be written by the editor of this JOURNAL, from data furnished by the subjects; and proof of each sketch will be sent to the subscriber, before printing, for approval. The work will also be edited by Dr. Daniel, who will have the entire supervision of its publication. There are a few subscribers who have not yet sent in their data; they must do so at once, or be left out. For a copy of the book address, inclosing price, the editor of this JOURNAL,—or the Texas Biographical Publishing Company.

